# Supplementary material for: Expression Profiles and Characteristics of Apple lncRNAs in Roots, Phloem, Leaves, Flowers, and Fruit
Source: Int J Mol Sci. 2022 May 25;23(11):5931. doi: 10.3390/ijms23115931 (PMC9180697; doi:10.3390/ijms23115931)
Supplement: Supplementary file 1 [file ijms-23-05931-s001.zip › Figure S.pdf]

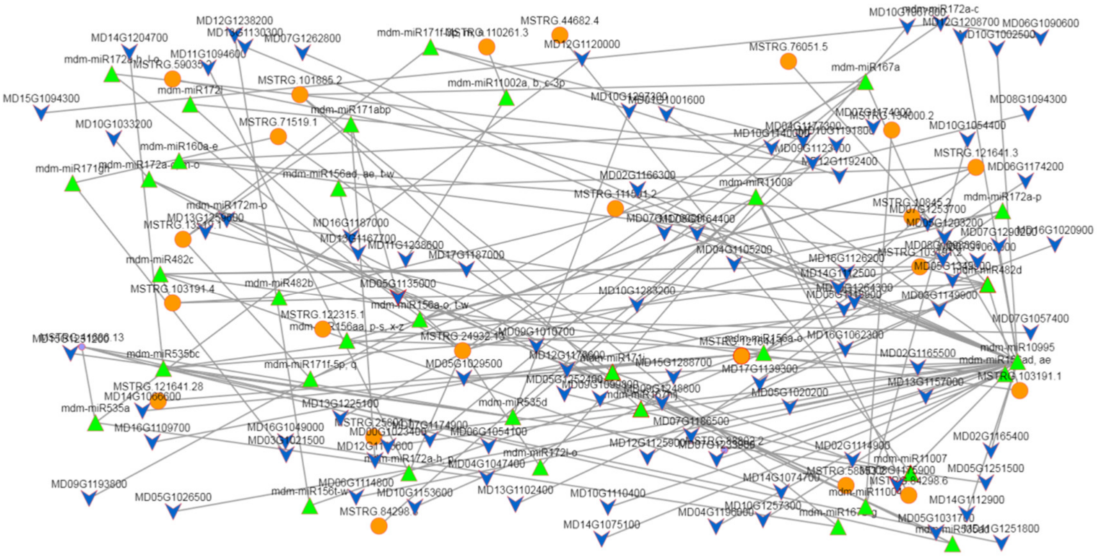

Figure S1. Potential lncRNA-miRNA-mRNA networks in all five tissues (orange circle: lncRNAs; green triangle: mdm-miRNAs; blue arrow: mRNAs).

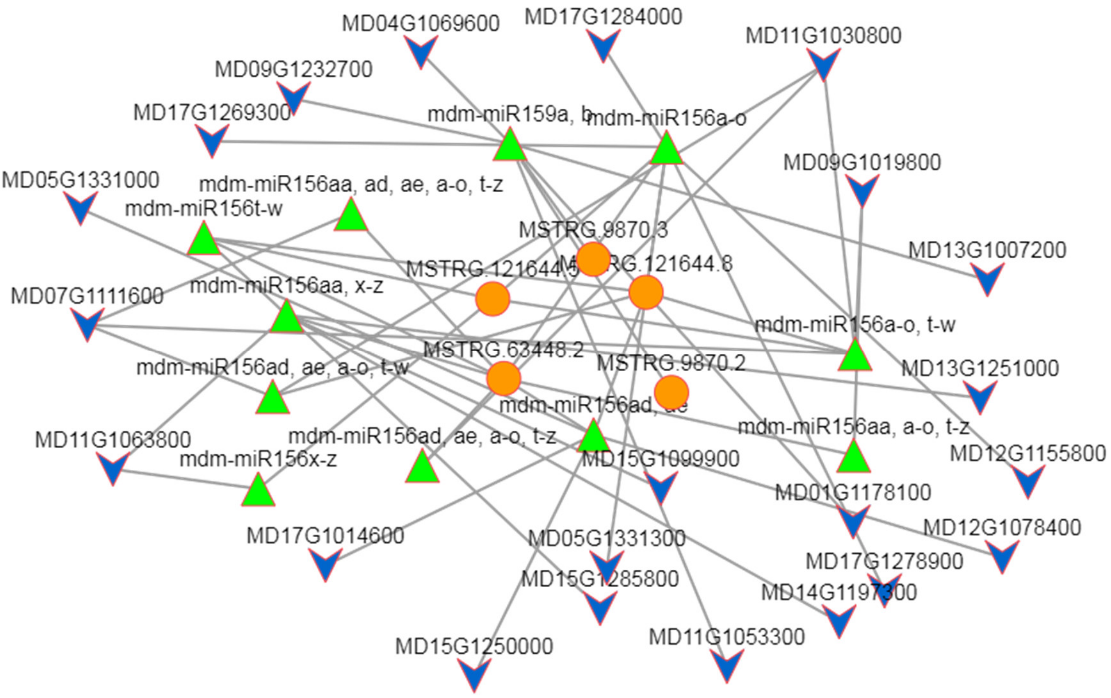

Figure S2. Potential lncRNA-miRNA-mRNA networks in roots only.

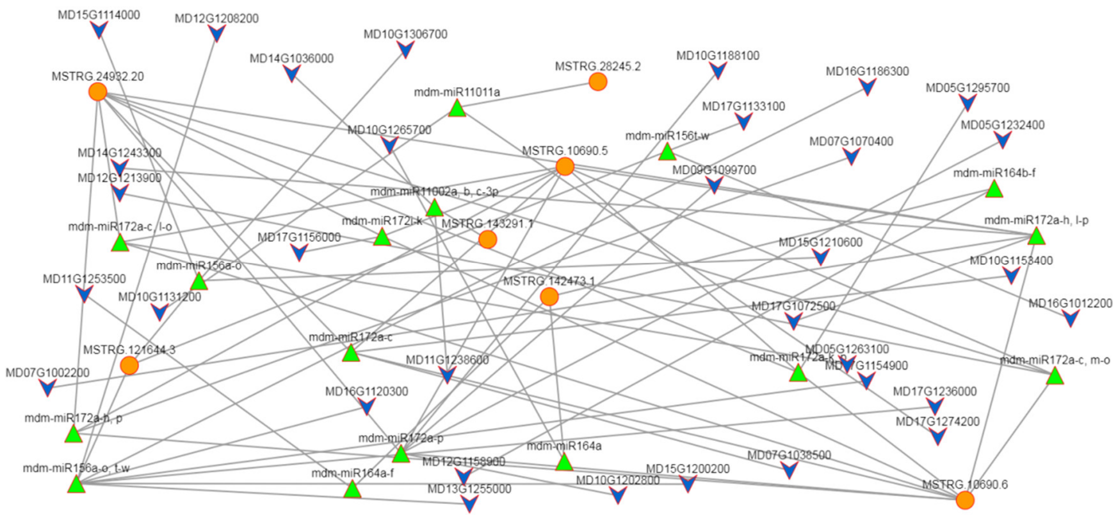

Figure S3. Potential lncRNA-miRNA-mRNA networks in leaves only.

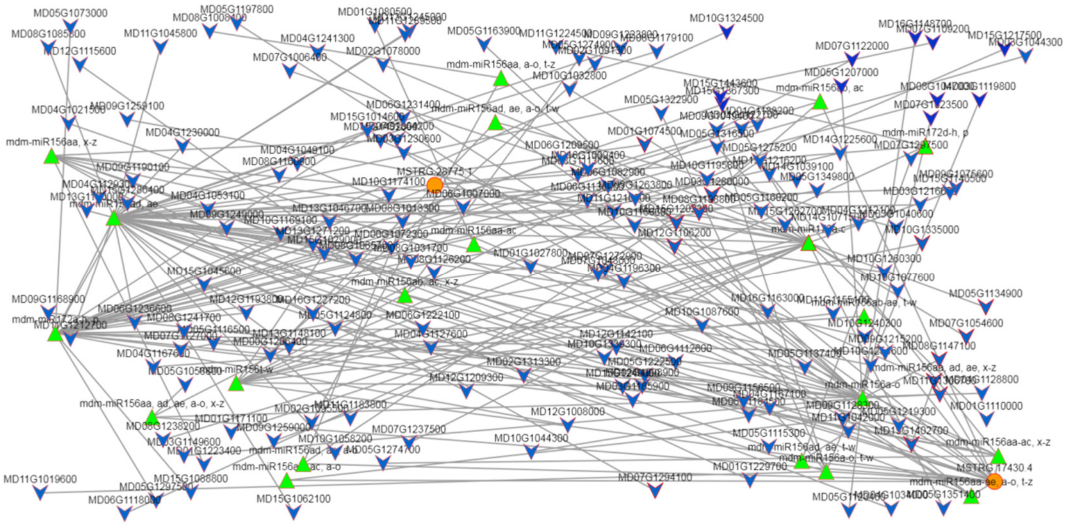

Figure S4. Potential lncRNA-miRNA-mRNA networks in flowers only.

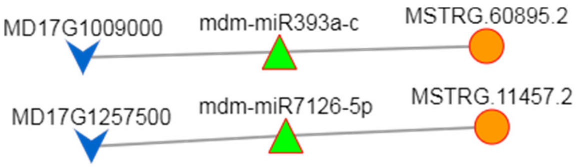

Figure S5. Potential lncRNA-miRNA-mRNA networks in fruit only.
